# Comparing two different superovulation protocols on ovarian activity and fecal glucocorticoid levels in the brown brocket deer (Mazama gouazoubira)

**DOI:** 10.1186/1477-7827-12-24

**Published:** 2014-03-19

**Authors:** Eveline S Zanetti, Marina S Munerato, Marina S Cursino, José Maurício B Duarte

**Affiliations:** 1 Deer Research and Conservation Center (NUPECCE, Núcleo de Pesquisa e Conservação de Cervídeos), Departamento de Zootecnia, Faculdade de Ciências Agrárias e Veterinárias, Universidade Estadual Paulista, Via de Acesso Professor Paulo Donato Castellane s/n, Jaboticabal, SP zip code: 14884-900, Brazil

**Keywords:** Reproduction biotechniques, Neotropical deer, Fecal glucocorticoids, Fecal progestin

## Abstract

**Background:**

Stress is a limiting factor in assisted reproduction in wild animals maintained in captivity. However, the knowledge of assisted reproduction techniques for wild animals is useful for future *in situ* and *ex situ* conservation programs. Thus, this study evaluated the ovulation rate, presence of functional corpora lutea and fecal glucocorticoid levels following treatments promoting superovulation in captive brown brocket deer.

**Methods:**

The crossover design used six hinds, allocated to two groups (n = 6): eCG Treatment, CIDR for 8 days, followed by 0.25 mg of EB on day 0, 700 IU of eCG on day 4 following device insertion and 265 mug of PGF2alfa on day 8; and FSH Treatment, CIDR for 7.5 days, followed by 0.25 mg of EB on day 0, 130 mg of FSH in 8 equal doses and 265 mug of PGF2alfa on day 7.5. Induced adrenal activity and treatment efficacy were evaluated by corpora lutea (CL) counts and fecal glucocorticoid and progestin concentration (ng/g feces) analyses for five different phases: Pre, two days before treatment; Early, first four days of treatment; Late, last four days of treatment; Total, entire treatment period; and Post, five days posttreatment.

**Results:**

eCG Treatment resulted in the highest number of CL (P lower than 0.05). There was no significant difference for fecal glucocorticoid concentrations in five different time periods between the treatments; however Pre fecal glucocorticoid concentrations (90.06+/−19.64) were significantly different from Late (200.76+/−26.39) within FSH Treatment. The mean fecal progestin concentration and mean ovulation rate were higher in eCG Treatment (4293.69+/−769.47, 7.0+/−1.8) than in FSH Treatment (1571.26+/−240.28, 2.6+/−0.8) (P lower than or equal to 0.05).

**Conclusions:**

Although the eCG Treatment induced a good superovulatory response, with the formation of functional corpora lutea, we cannot yet affirm that we have established a suitable protocol for induction of SOV in the species *M. gouazoubira* because approximately 65% of the deer showed premature regression of the corpora lutea. Moreover, multiple FSH applications in FSH Treatment resulted in a low ovulation rate and induced an increase in fecal glucocorticoid levels.

## Background

The brown brocket deer (*Mazama gouazoubira*) is one of the most abundant species in the Neotropical region [1] and is classified as LC (least concern) in the IUCN Red List [2]. It is considered a monovulatory nonseasonal breeder, giving birth throughout the year [3,4]. Data from endocrinology studies, based on progestogen data, indicate a mean luteal phase duration of 24.6 ± 1.4 day and a mean interluteal phase duration of 1.7 ± 0.1 day [3]. It is also one of the most studied species regarding the endocrinology of the estrous cycle and pregnancy [3] and the development of reproductive biotechnologies, such as estrus synchronization [5,6] and superovulation [5,7].

The abundance of this species and the preceding studies, which increased knowledge concerning the species, favor its use as an experimental model to evaluate reproductive techniques that could be used as a starting point for other neotropical deer species [7], since 59% of these species are classified as vulnerable or threatened with extinction [2].

Among the available biotechnologies, stimulation of follicular growth to induce superovulation is essential to the success and efficiency of embryo transfer and in vitro fertilization programs [8], techniques that permit complete flexibility in sire/dam pairings [9]. Although multiple ovulation has been performed successfully in brown brocket [7], red (*Cervus elaphus*[8-17], fallow (*Dama dama*[11,13,18]), Pere David’s (*Elaphurus davidianus*[15]), white-tailed deer (*Odocoileus virginianus*, [19]), and some other domestic species [20,21], the procedure often results in highly variable superovulatory responses [10,20,21] and thus it is the weakest link in the chain of events required for successful multiple ovulation/embryo transfer technology (MOET) [22].

The wide variation in the superovulatory response could be related to numerous factors, such as the status of follicular development of the deer at the time superovulatory treatment was initiated [20], age, weight, nutrition, season, the type and combination of gonadotropins used and genetic factors [23]. Moreover, the stress induced by intense manipulation of the deer during assisted reproductive procedures is a particularly important factor in wild animals and could explain the poor responses obtained in wild ungulates [24]. Multiple ovulation protocols involve a single injection of equine chorionic gonadotropin (eCG) and/or multiple injections of follicle stimulating hormone (FSH) [13] and should be designed to eliminate as much handling stress as possible [25].

Several studies of domestic and wild animals have discussed the influence of stress on reproductive function in females [24,26-28] and one widely used method to determine this in mammals is the evaluation of adrenal gland activity in the production of catecholamine and glucocorticoids, particularly the latter, which are commonly used as indicators of biological neuroendocrine response to stress [29]. For this reason, many researchers and conservationists have begun to use a noninvasive method, involving indirect measurement of glucocorticoid metabolites as a means to determine stress in several animal species [30], since blood collection for plasma glucocorticoid quantification is known to cause stress, especially in wild animals, disturbing the final outcomes of the analysis [31].

The quantification of fecal glucocorticoids has been used to evaluate the influence of husbandry systems on the physiological stress of captive brown brocket deer [32]; however, to the best of our knowledge, no information is available concerning the consequences of stress on superovulation protocols in this species or in other neotropical cervids. Thus, the aim of this study was to evaluate the ovulation rate, the presence of functional corpora lutea and fecal glucocorticoid levels following two distinct treatments designed to promote superovulation in captive brown brocket deer.

## Methods

This study was approved by the Animal Ethics and Welfare Committee (*Comitê de Ética e Bem-estar Animal*, CEBEA) of the Faculty of Agrarian and Veterinary Sciences (*Faculdade de Ciências Agrárias e Veterinárias*, FCAV) UNESP, Jaboticabal, SP, Brazil (protocol number 013147–06, 02-08-2006).

### Animals

Six adult hinds (primiparous, aged 2.0 to 4.0 years-old, weighing 12.9 to 20.6 kg) and 1 male (vasectomized, 3.0 years-old, weighing 18.0 kg) were housed at the Deer Research and Conservation Center (NUPECCE) facilities at the São Paulo State University (UNESP), Jaboticabal Campus (20°S latitude). From March through August 2009, all the deer were maintained individually in stalls (3 m × 2 m) with auditory and olfactory contact with conspecific males and females and were exposed to normal fluctuations in the photoperiod. They were fed *ad libitum* with a diet consisting of a pelleted ration (12% crude protein, 2% crude fat, 10% crude fiber, Purina Co.; Paulínia, São Paulo, Brazil) and approximately 1 kg/deer/day of fresh alfalfa (*Medicago sativa*), perennial soybean (*Neonotonia wightii*) or mulberry branches (*Morus alba*). Water was also provided *ad libitum*.

### Estrus synchronization and superovulation treatment

**Figure 1:**
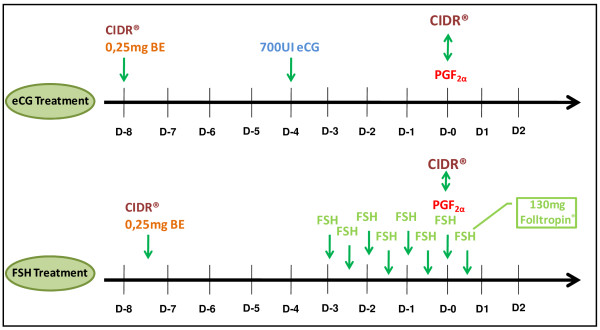
**Two superovulation treatments performed on six hinds of the species *Mazama gouazoubira*: eCG Treatment (CIDRÒ for 8 days + EB D-8 + 700UI eCG D-4 + cloprostenol D0) and FSH Treatment (CIDRÒ for 7.5 days + EB D-7.5 + 130 mg FSH D-3 / D0.5 + cloprostenol D0).**

Initially, on a random day of the estrous cycle, the hinds were divided into two groups (n = 6): (i) eCG Treatment, in which the hinds received a single intravaginal device containing 0.33 g of progesterone (CIDR-type T; Controlled Internal Drug Release; Pfizer, USA) for eight days [6], followed by 0.25 mg i.m. injection of estradiol benzoate (EB) (0.25 mL Estrogin; Farmavet Produtos Veterinários Ltda, Brazil) [33,34] on the same day as the device was inserted (to induce atresia in ovarian follicles) [20], 700UI i.m. injection of eCG (3.5 mL Folligon 1000; Intervet International BV; Holland) on day 4 following device insertion (to induce superovulation, SOV) and 265 μg i.m. injection of PGF_2α_ (265 μg cloprostenol; 1 mL Ciosin; Schering Plough Coopers, Brazil) on the day the device was removed, to ensure that any persistent luteal tissue was removed upon progesterone withdrawal [6]; (ii) FSH Treatment, in which the hinds received a single intravaginal device containing 0.33 g of progesterone for 7.5 days, followed by 0.25 mg i.m. injection of EB on the same day as the device was inserted, 130 mg i.m. of FSH (6.5 mL Folltropin-V; Bioniche Animal Health; Canada) applied in 8 equal doses, such that the first dose was administered on day 4.5 post-insertion of the device and the last dose was administered 12 h following its removal, and 265 μg i.m. injection of PGF_2α_ on the day the device was removed (Figure 1). Each hind received the treatments in a cross-over design; all the deer were submitted to both treatments [35]. The interval between the treatments was 44–48 days following the end (removal of the intravaginal device, CIDR®) of the first treatment which the hind was submitted. This experimental design was adopted in an attempt to control temporal effects, to reduce the effect of one treatment on the other, to control hind variability, while evaluating treatment effects, and because of the limited number of experimental animals available and the difficulties involved in handling wild deer. Only insertion of the CIDR® progesterone device and the EB applications were performed during physical restraint [36] between 8:00 and 9:30 a.m. To perform the other procedures, the hinds were led to a restraint box. All the hinds received a second i.m. injection of 265 μg of cloprostenol 14 days after the removal of the CIDR® device used to induce luteolysis of multiple corpora lutea (CL).

### Assessment of ovarian stimulation

The ovulation rate was evaluated by CL counts and total follicular stimulation was evaluated by CL and counts of large (over 3 mm [6]) unruptured follicles seven days after the first copulation [22] (or day 9 or 10 following the completion of treatment for deer that showed no behavioral estrus) by laparoscopy, such that hinds with two or more CL were defined as having superovulated. The procedure involved with holding food and water from the hinds for 24 h and physical restraint, followed by anesthesia, as previously described for *M. gouazoubira*[6]. The laparoscopic procedures used were the same as those described for red deer [12]. The incisions were closed with 2–0 nylon suture and the hinds were treated with benzathine penicillin (Pentabiotic; Fort Dodge, Campinas, Brazil, 40,000 IU/Kg i.m.) and monophenylbutazone (Monofenew; Vetnil, Louveira, Brazil, 5 mg/Kg, i.v.).

### Behavioral data and sample collection

Signs of estrous behavior were determined by allowing each hind to associate with a vasectomized male adult (10 min every 12 h) from 12 h after the completion of the treatments until the moment that the hind no longer accepted copulation (end of estrus). Behavioral estrus was defined as the period in which hinds permitted copulation [3]. Fecal samples were collected daily (between 7:30–9:30 h) from two days prior to the onset of treatment (progesterone device insertion) until five days after the completion of the treatment (progesterone device removal) and from the day of laparoscopy (day 7 after first copulation or day 9 or 10 after the completion of treatment for deer that showed no behavioral estrus) until the final (second) application of cloprostenol (day 14 following removal of the device). Fecal samples were also collected on day 17 following removal of the device. Each sample recovered within 12 h of voiding was placed in individually labeled plastic bags. The fecal samples were frozen (−20°C) within 20 min of collection. All the samples were stored at -20°C until steroid analysis was performed.

### Fecal steroid extraction and enzyme immunoassays (EIA)

The fecal samples (total amount collected) for each treatment were dried in an oven (Mod. 320-SE, Fanem Ltda., São Paulo, Brazil) at 56°C for approximately 72 h [37]. The dried fecal samples were pulverized and the steroids were extracted from the feces according to the method described by Graham et al. [38]. A proportion of the resulting powder (0.5 g) was weighed and extracted with 5 mL of 80% methanol. After vortexing for 30 s at high speed, the sample was shaken for 12 h on a mechanical shaker. Following centrifugation at 375 g for 20 min, the supernatant was transferred into a clean tube. Aliquots of supernatant were diluted, 1:250 for the progesterone assay and 1:8 for the cortisol assay, for EIA analyses (Multiskan MS, Labsystem, Helsinki, Finland). The concentrations were determined using CL425 and R4866 antibodies (C.J. Munro, University of California; Davis, CA, USA) for progestogens and glucocorticoid, respectively. These antibodies were chosen due to their high cross-reactivity with the metabolites excreted in *M. gouazoubira* feces, 5α- and 5β-pregnanes and cortisol [39]. Validation of the hormone dosages was performed according to Brown et al. [40], by observation of the parallel disposition between the standard curve and that formed by the pool of fecal extracts prepared by serial dilution and by substantial recovery of exogenous progesterone (y = 0.99× + 1.14, r^2^ = 0.98) and cortisol (y = 0.8664× + 21.20, r^2^ = 0.99) added to fecal extracts. Interassay coefficients of variation for two separate controls were 4% (30% binding) and 17% (70% binding) for fecal progestogens metabolites and 9% (27% binding) and 14% (69% binding) for fecal corticosteroids metabolites. Intraassay coefficients of variation were <10%. Physiological validation to assess the physiological relevance of measuring fecal glucocorticoid metabolites (FGM) in *M. gouazoubira*, has already been determined by Christofoletti et al. [32]. All fecal data are expressed on a dry-weight basis.

### Statistical analysis

For analysis of data presented as mean ± standard error of the mean (SEM), the Student t test for independent samples was used. Nonparametric data (presence or absence of superovulation, presence or absence of behavioral estrus and regression or not regression of CL) were analyzed using the Fisher Exact test. To assess the significance of the differences between means for number of CL in the right or left ovary within each treatment, the Student t test for dependent samples was used. The fecal hormone concentration values were submitted to analysis of variance following logarithmic transformation of the hormone data [41]. For analyses of the mean FGM, each treatment was divided into five different phases: Pre, two days before treatment; Early, the first four days of treatment; Late, the last four days of treatment; Total, the entire treatment period; and Post, five days after treatment. To examine the effects of these five phases on FGM, within each treatment, repeated measures analysis of variance (ANOVA) was used, followed by the Tukey multiple comparison test. To examine the effects of treatment on FGM, the Student t test for independent samples was used. The mean fecal progesterone metabolites (FPM) produced by the CL (corpora lutea, luteal phase), was calculated from the mean FPM of each of the hinds, between laparoscopy (day 7 after first copulation or day 9 or 10 after the completion of treatment for deer that showed no behavioral estrus) and the final (second) application of cloprostenol (day 14 following removal of the device); the mean and SEM were calculated based on the results of four values from eCG Treatment and five values from FSH Treatment. Hinds 3 and 5 were excluded from the mean of anovulatory follicles, total follicle stimulation, number of CL and luteal FPM for eCG Treatment, while hind 3 has was excluded from the same means for FSH Treatment due to the repetition of estrous behavior before the laparoscopic examination. To compare the mean FPM during the luteal phase, on days 14 and 17 (following removal of the device), between treatments, the Student t test for independent samples was used to analyze the effects of the treatments on FPM. To compare FPM on days 14 and 17 (following removal of the device), within each treatment, the Student t test for dependent samples was used to determine the effects of days on FPM. Hinds 2 and 5 were excluded from the FPM means on days 14 and 17 in both treatments due to the repetition of estrous behavior in the period between the laparoscopic procedure and day 14 following removal of the CIDR® device. All hormones analyzes were performed considering the delay time (24 h) between hormonal events in the plasma and the appearance of the respective signal in the feces. The Pearson correlation test was used to correlate the FGM from the Late Treatment phase with FPM produced by CL and the number of anovulatory follicles. Correlations were analyzed using combined data from both treatments. All analyzes were performed using Minitab® 14 (Minitab Inc., State College, Pennsylvania State University, USA). Values were considered significant when P < 0.05.

## Results

### Estrus and ovarian response

The results for estrus and superovulatory response are presented in Table 1. The number of hinds in estrus, superovulated hinds, the interval between the completion of treatment and the onset of estrus and duration of estrus did not differ between the treatments (P ≥ 0.05). The period between the completion of treatment and the onset of estrus ranged from 24–48 h and for eCG Treatment and 48–120 h for FSH Treatment. Thus, synchrony, i.e. the interval between the first and last female showing the estrous behavior, was better in eCG Treatment than in FSH Treatment.

**Table 1:** **Mean (±SEM) time to estrus and superovulatory response in six hinds of the species *Mazama gouazoubira *** **submitted to two different hormonal treatments**

	**Treatments**		**P value**
	**eCG**	**FSH**	
**Number of hinds treated per treatment**	6	6	
**Hinds exhibiting estrus behavior**	3/6	3/6	NS
**Period between removal of the progesterone device and the onset of estrus (h) (range)**	40.0 ± 8.0 (24–48)	92.0 ± 22.0 (48–120)	NS
**Duration of estrus (h) (range)**	40.0 ± 4.0 (36–48)	36.0 ± 6.9 (24–48)	NS
**Synchrony**^**a **^**(h)**	24	72	
**Superovulating hinds**	4/6	4/6	NS
**No. of corpora lutea (range)**	7.0 ± 1.8a* (2–10)	2.6 ± 0.8b* (1–6)	0.04
**No. of corpora lutea in the right ovary (range)**	4.2 ± 1.2* (1–7)	1.0 ± 0.3* (0–2)	NS
**No. of corpora lutea in the left ovary (range)**	2.7 ± 0.8* (0–5)	1.6 ± 0.8* (0–2)	NS
**No. of anovulatory follicles (range)**	4.5 ± 1.7* (0–7)	7.6 ± 0.9* (6–11)	NS
**Total follicular stimulus**^**b **^**(range)**	11.5 ± 2.5* (6–17)	10.2 ± 1.1* (7–13)	NS
**Hinds with early regression of corpora lutea**	4/6	2/6	NS

eCG Treatment, CIDR^®^ for 8 days + BE D0 + 700UI eCG D4 + cloprostenol D8; and FSH Treatment, CIDR^®^ for 7.5 days + BE D0 + 130 mg FSH D4.5/D8 + cloprostenol D7.5.

^a^Period between the first and last hind to exhibit estrus behavior under each treatment.

^b^Sum of the number of corpora lutea and the number of anovulatory follicles.

*Mean ± SEM, excluding data from hinds 3 and 5 in eCG Treatment and hind 3 in FSH Treatment due to repetition of estrus behavior prior to laparoscopic examination.

NS: *P ≥ 0:05*; Values with different letters (a and b) along the lines differ (P < 0:05) according to the Student t test.

Regarding ovulatory response, eCG Treatment resulted in the highest number of CL (P < 0.05). However, the mean rate of anovulatory follicles and total ovarian stimulation were not significantly different between treatments (Table 1). In addition, no significant differences were verified between the number of ovulations in the right and left ovaries within each treatment.

Hind 5 did not present estrous behavior immediately following completion of the eCG Treatment, but on the day of the laparoscopic examination (10 days after removal of the CIDR® device), during which, the presence of two CL, two CL in regression and two large ovulatory follicles were observed. This treatment also revealed the presence of corpora albicantia and CL with signs of regression in hinds 2 and 4. These deer presented estrous behavior 12 and 14 days after the completion of eCG Treatment, respectively. Estrous behavior was not observed in hind 4 immediately following the removal of the CIDR® device.

In eCG and FSH Treatments, hind 3 presented repetition of behavioral estrus two days before the laparoscopic examination (7 days after the completion of treatment). During the exam, the presence of CL, large follicles and small diffuse follicles were all observed. CL showing signs of regression were also observed for hind 4 in FSH Treatment.

Hinds 2, 3, 4, 5 and 6 presented behavioral estrus (lasting up to 3 days) following the administration of EB and continued to cycle in the period between treatments. With the exception of hinds 2, 4 and 5 in eCG Treatment and hinds 2 and 5 in FSH Treatment, all the other deer showed behavioral estrus following the final administration of cloprostenol (14 days after the removal of the CIDR® device).

### Endocrine profiles

**Figure 2:**
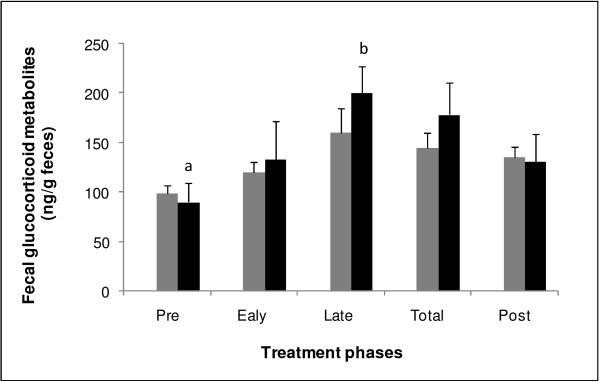
**Mean (±SEM) fecal glucocorticoid metabolites (ng/g feces) of six brown brocket deer (*Mazama gouazoubira*) hinds for five different phases (Pre, two days before treatment; Early, the first four days of treatment; Late, the last four days of treatment; Total, entire treatment period; and Post, five days after treatment) of two treatments: eCG, CIDRÒ for 8 days + BE D0 + 700UI eCG D4 + cloprostenol D8, gray bars; and FSH, CIDRÒ for 7.5 days + BE D0 + 130 mg FSH D4.5 / D8 + cloprostenol D7.5, black bars.** Means within FSH Treatment with different letters (**a** and **b**) differ (P < 0.05) according to the Tukey test.

The mean FGM during the five different phases of eCG and FSH Treatments are shown in Figure 2. No significant differences in FGM concentration were determined between the treatments for the five different phases; however, the Pre treatment FGM concentration was significantly different from the Late treatment in FSH Treatment.

Regarding the two SOV protocols, the mean FPM produced by the CL (luteal phase) was higher (P < 0.05) in eCG Treatment than in FSH Treatment (Table 2). Administration of cloprostenol 14 days after removal of the CIDR® device was associated with the demise of luteal tissue, as indicated by the abrupt decline (P < 0.05) in mean FPM in eCG Treatment 72 h later (Table 2). A fall in mean FPM in FSH Treatment also occurred, though this was not significant.

**Table 2:** **Mean (±SEM) fecal progesterone metabolites [FPM] of hinds of the species *Mazama gouazoubira*, in the different phases of two hormonal treatments**

	**Treatments**	
	**eCG**	**FSH**
**[FPM] luteal**^**a **^**(ng/g feces)**	4293.69 ± 769.47a*	1571.26 ± 240.28b*
**[FPM] pre-PGF**^**b **^**(ng/g feces)**	4002.76 ± 11019.48Aa**	1296.09 ± 175.69Aa**
**[FPM] post-PGF**^**c **^**(ng/g feces)**	1147.18 ± 267.40Ba**	424.82 ± 104.37Aa**

1, CIDR^®^ for 8 days + EB D0 + 700UI eCG D4 + cloprostenol D8; and 2, CIDR^®^ for 7.5 days + BE D0 + 130 mg FSH D4.5/D8 + cloprostenol D7.5.

^a^[FPM] in the period between laparoscopy and day 14 following removal of the CIDR^®^ device; ^b^[FPM] on day 14 following removal of the CIDR^®^ device/the day of the final PGF_2α_ injection; ^c^[FPM] on day 17 following removal of the CIDR^®^ device /3 days after the final PGF_2α_ injection; *Mean ± SEM, excluding data from hinds 3 and 5 in eCG Treatment and hind 3 in FSH Treatment due to repetition of estrus behavior prior to laparoscopic examination.

**Mean ± SEM, excluding data from hinds 2 and 5 in eCG and FSH Treatments due to repetition of estrus behavior in the period between laparoscopic examination and day 14 following removal of the CIDR^®^ device. NS: P ≥ 0.05; Means within each column with different capital letters (A and B) differ (P < 0.05) according to the paired Student t test; Means within each row with different lowercase letters (a and b) differ (P < 0.05) according to the Student t test.

### Correlations

The correlation analysis showed a positive correlation between FGM and number of anovulatory follicles (0.52, P ≥ 0.05), and a negative correlation between FGM and FPM (−0.12, P ≥ 0.05).

## Discussion

Our analysis of the results of this study show that the two proposed treatments (eCG Treatment, CIDR® for 8 days + EB D0 + 700UI eCG D4 + cloprostenol D8; and FSH Treatment, CIDR® for 7.5 days + EB D0 + 130 mg FSH D4.5/D8 + cloprostenol D7.5) were capable of promoting a superovulatory response; however, eCG Treatment produced the greatest number of corpora lutea, while FSH Treatment promoted only a minimal response (2.6 ± 0.8 CL), sufficient to be considered superovulated.

The best mean ovulation obtained in eCG Treatment (7.0 ± 1.8 CL) was similar to the means obtained in the species *D. dama* (6 to 10 CL [11,13,18]) and *C. elaphus*, which have already been selected for MOET programs (mean of 5 to 12 CL [10–13,15-17 ]). Moreover, this mean was higher than the ovulation means obtained for other species, such as *E. davidianus* (CL 3.83 [15]), *O. virginianus* (3.10 CL [19]) and *C. elaphus*, which were not selected for a MOET program (2.15 CL [14]). Higher mean ovulation (3.40 ± 0.68 CL) has already been obtained for the species *M. gouazoubira*, in a previous experiment which used similar SOV protocols with modifications in the EB dosage (0.5 mg) used to induce ovulatory follicular atresia and synchronize the follicle wave in conjunction with the CIDR® and eCG (600UI) used to induce SOV [7].

These two modifications in the protocol could begin to explain the differences between the ovulatory rates obtained in eCG Treatment and in the previous study with the same species. The use of a lower dose of EB and a higher dose of eCG seem to have been a positive factor. It has already been shown that high levels of estradiol at the moment of insertion of the progesterone implant and early administration of gonadotropins have a negative impact on superovulatory response and on follicular wave synchronization [23,42] and that the ovulation rate increases in a dose-dependent manner [43] in relation to an increase in the eCG dosage [7]. However, even using a lower dose of EB, it is probable the protocol is not yet well adjusted to the needs of this species, since the hinds presented behavioral estrus following the administration of EB. Another factor that could explain the lower ovulation rate obtained in the previous study are changes in luteinizing hormone (LH) pulsatility caused by stress induced by manual physical restraint [44] during the hormone applications, which in this present study were substituted by leading the deer to a restraint box.

Although considered low, the ovulation rate obtained in FSH Treatment was three times higher than the rate obtained in a previous study (0.80 ± 12:49 CL) involving the species *M. gouazoubira*, which used 250 IU FSH (Pluset®, Calier®, Spain) administered in a single application, together with a synthetic organic polymer (polyvinylpyrrolidone), used in the processing of long-acting drugs [7]. The regime of multiple FSH applications seems to be the most suitable for stimulating SOV in cervids; however, despite the way it was conducted, it still seems inadequate to provide good ovulatory stimulus, since the total follicular stimulation of eCG and FSH Treatments were similar, but the ovulation rate of eCG Treatment was approximately 2.5 times greater than that for FSH Treatment.

In general, the system of multiple FSH applications is influenced by the effects of stress management [24,25], as suggested by the significant increase in FGM concentrations after day 4 of FSH Treatment, when the FSH applications were initiated. The FGM levels increased during period of intensive management of the deer, as previously reported for *Gazella dama mhorr*[24]. Although the FGM levels of FSH Treatment were higher, no significant difference was verified between the treatments, which may indicate that only one application of eCG on day 4 was sufficient to increase FGM concentrations and that the use of eCG provided extra gonadotrophic stimulation for ovulation [45] in eCG Treatment, as reported in studies with other deer species that combine low doses of eCG at the end of treatments with FSH [45] due to the lack of SOV response when FSH was used alone [14]. The reinforcement of eCG on the preovulatory peak in eCG Treatment can also be inferred from shorter synchrony period of this treatment, suggesting that stimulation of ovulation of a greater number of follicles occurred in a shorter period of time [45].

The use of eCG has been associated with the occurrence of asynchronous follicular development and anovulatory follicles in numbers proportional to the doses used [43]. However, the occurrence of anovulatory follicles in this study does not seem to be exclusively related to the use of eCG, since FSH treatment also resulted in numerous anovulatory follicles. The high levels of FGM during the follicular growth phase should also be considered as an important factor in the occurrence of these follicles, due to endocrine disorders caused by the release of corticosteroids [46] and/or progesterone production by the adrenal gland [47], which can inhibit the LH peak and/or affect oocyte competence [24], resulting in lower receptor acquisition and, consequently, lower responsiveness to LH [48].

Other factors, including the high concentrations of estradiol secreted by anovulatory follicles, can also significantly reduce the rates of ovulation and total ovarian stimulation, as well as promoting premature regression of approximately 10% of the CL, similar to that previously reported for *C. elaphus*[8,16,49] and other domestic species [21,50]. Although both treatments resulted in a large number of anovulatory follicles, in eCG Treatment, four hinds (66.7%) presented repetition of behavioral estrus and signs of premature regression of CL, whereas in FSH Treatment, only two hinds (33.3%) showed the same signs, suggesting that premature regression is more closely associated with the use of eCG and its prolonged action than the mere occurrence of anovulatory follicles.

In addition to these factors, it is important to consider that the ovulation rates obtained in this study may have been influenced by the administration of gonadotropins at an inappropriate time in the follicular wave, since the first study that induced follicular wave emergence in cervids was developed in *C. elaphus*, during anestrus, and demonstrated the onset of a new wave 5.2 ± 0.2 days following the administration of progesterone and estradiol-17β [34]. Moreover, the use of an ovulation inductor could have improved the ovulation rates and should be considered in future protocols.

The profiles of FPM concentration during the luteal phase associated with the surgical view of corpora lutea revealed a luteal source of secretion following by ovulation in both treatments. The increase in the ovulation rate was associated with increased progesterone secretion as a result of the significant increase in luteal tissue mass [7,13]. The secretion of progesterone during this period was apparently not influenced by the secretion of glucocorticoids in the final phase of the treatments, as confirmed by the correlation value obtained and as previously reported by González et al. [24]. However, this result could have been influenced by the production of progesterone in the adrenal gland [47] and/or individual variations among the small sample of deer used in the study.

As previously reported for the species *M. gouazoubira*[7], administration of cloprostenol 14 days after the completion of treatment resulted in rapid destruction of luteal tissue and a return to baseline concentrations of progesterone within 72 hours. This fact is relevant in embryo transfer programs to prevent the implantation of embryos that may remain following collection and to resynchronize the females for natural mating [13].

## Conclusions

In conclusion, our results indicate that although the eCG Treatment (700UI eCG) induced a good superovulatory response, with the formation of functional corpora lutea, we cannot yet affirm that we have established a suitable protocol for induction of SOV in the species *M. gouazoubira* because approximately 65% of the deer showed premature regression of the corpora lutea. Moreover, multiple FSH applications in FSH Treatment resulted in a low ovulation rate and induced an increase in FGM levels.

## Abbreviations

CL: Corpora lutea; CIDR: Controlled internal drug release; EB: Estradiol benzoate; eCG: Equine chorionic gonadotropin; EIA: Enzyme immunoassays; FGM: Mean fecal glucocorticoid metabolites; FPM: Mean fecal progesterone metabolites; FSH: Follicle stimulating hormone; LH: Luteinizing hormone; NUPECCE: Deer research and conservation center; PGF2α: Cloprostenol; SEM: Standard error of the mean; SOV: Superovulation.

## Competing interests

The authors declare that there are no competing interests.

## Authors’ contributions

ESZ and JMBD contributed to design the study and analyzed the data. ESZ, JMBD, MSM and MSC contributed during the experimental phase. All authors have contributed to drafting paper. All authors read and approved the final manuscript.
